# Solid-Phase Synthesis of New Trp(Nps)-Containing Dipeptide Derivatives as TRPV1 Channel Blockers

**DOI:** 10.3390/molecules15074924

**Published:** 2010-07-15

**Authors:** Mª Jesús Pérez de Vega, Mª Teresa García-López, Laura Zaccaro, Miriam Royo, Fernando Albericio, Asia Fernández-Carvajal, Antonio Ferrer-Montiel, Rosario González-Muñiz

**Affiliations:** 1 Instituto de Química Médica, CSIC, Juan de la Cierva, 3, 28006 Madrid, Spain; 2 Combinatorial Chemistry Unit, Barcelona Science Park, University of Barcelona, Barcelona 08028, Spain; 3 Institute for Research in Biomedicine, Barcelona Science Park, University of Barcelona, Barcelona 08028, Spain; 4 CIBER-BBN, Networking Centre on Bioengineering Biomaterials and Nanomedicine, Barcelona 08028, Spain; 5 Department of Organic Chemistry, University of Barcelona, Barcelona 08028, Spain; 6 IBMC-UMH, Ed. Torregaitán, Av. de la Universidad s/n, 03202 Elche, Spain

**Keywords:** solid-phase, Trp(Nps) dipeptides, TRPV1 channel blockers

## Abstract

Trp(Nps)-Lys-NH_2_ derivatives, bearing alkyl or guanidine groups either at the *N*–terminus or on the Lys side-chain or at both positions were conveniently prepared on solid-phase and evaluated as TRPV1 channel antagonists.

## 1. Introduction

TRPV1 (Transient Receptor Potential Vanilloid 1) is a non-selective, Ca^2+^ preferring, ion channel, activated by temperatures higher than 42 ºC, acidic pH, vanilloids such as capsaicin, and the endogenous cannabinoid receptor ligand anandamide [1]. TRPV1 expression is up-regulated in a number of painful disorders, including chronic, neuropathic and acute inflammatory pain, and consequently, it is a promising therapeutic target for pain relief [1,2]. In this respect, numerous research programs have been dedicated to the search of TRPV1 modulators to be used as pharmacological tools for better understanding the pharmacology of this cation channel. In addition, few modulators that emerged from these programs have reached clinical trials for multiple therapeutic indications [3,4]. However, due to possible side effect issues, such as diminished response to damaging heat stimuli, altered body temperature and reduction in the perception of taste, the development of TRPV1 antagonists as analgesic drugs still needs compounds with greater efficacy and fewer adverse effects [3,4].

Like ruthenium red [5], Arg-rich peptides have been described as non-competitive antagonists of TRPV1 receptors, through binding to a site located near the entryway of the aqueous pore [6]. A few years ago, we found that dipeptide derivatives Xaa-Trp(Nps) and Trp(Nps)-Xaa (Xaa=Lys, Arg, Nps = 2-*o*-nitrophenylsulfenyl), first described as analgesic dipeptides of unknown mechanism of action [7,8], inhibited the activation of TRPV1 in the micromolar range, and also acted as NMDA channel blockers, although with lower potency [9]. In view of their structural analogy with the Arg-rich peptides, it was hypothesized that these Nps-containing dipeptides could interact with the vanilloid receptor through the pore entrance. Now, in an attempt to fine-tune the potency/selectivity balance within this family of channel blockers, we have prepared and evaluated a new series of H-Trp(Nps)-Lys-NH_2_ dipeptide derivatives, incorporating alkyl and guanidino moieties at the *N*–terminal group and on the Lys side-chain. This paper deals with the solid-phase synthesis and the biological activity displayed by this new series of Trp(Nps)-containing dipeptides, compared to model compounds H-Trp(Nps)-Lys-NH_2_ (**1**) and H-Trp(Nps)-Arg-NH_2_ (**2**).

## 2. Results and Discussion

### 2.1. Synthesis

**Scheme 1:**
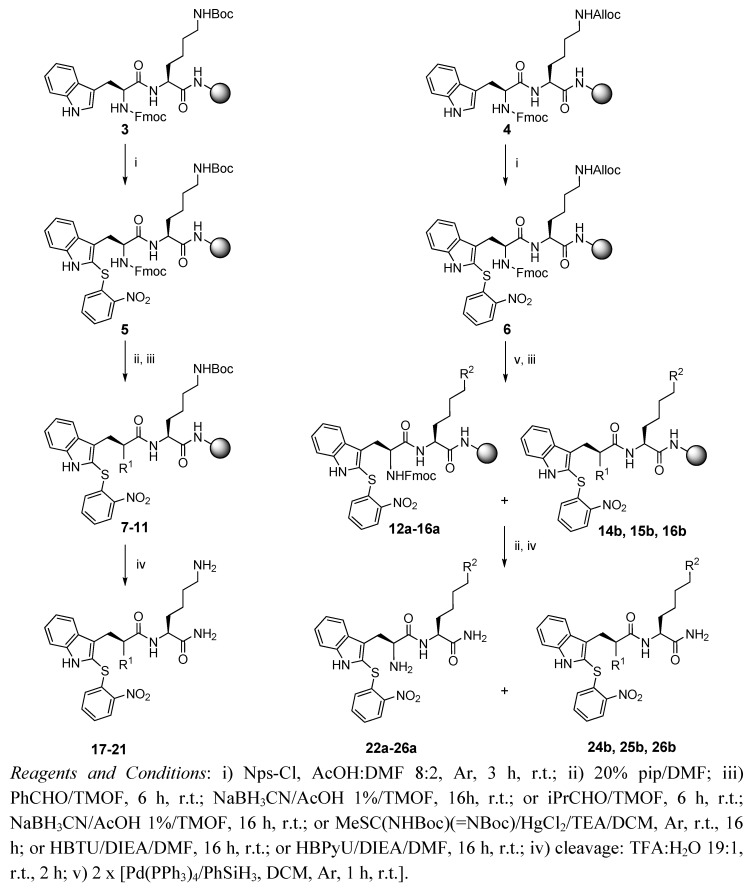
Synthesis of Trp(Nps)-containing dipeptide derivatives.

The synthesis of the Trp(Nps)-Lys-NH_2_ dipeptide derivatives was performed by parallel solid-phase synthesis on a Rink-amide MBHA polystyrene resin (NH_2_-PS), following a Fmoc/*^t^*Bu strategy (Scheme 1). Fmoc-Trp-Lys(Boc)-NH-PS (**3**) and Fmoc-Trp-Lys(Alloc)-NH-PS (**4**) were prepared from the corresponding amino acid derivatives as appropriate intermediates for modifications at the N–terminus and the Lys side-chain, respectively. Then the most demanding step, the incorporation of the Nps moiety, was performed by reaction with 2-nitrobenzenesulphenyl chloride (Nps-Cl) in an 8:2 AcOH-DMF mixture, to produce dipeptidyl resins **5** and **6**. After the orthogonal removal of the Fmoc group of **5**, two portions of the resin were subjected to reductive amination by condensation with benzaldehyde and isobutylaldehyde, followed by treatment with NaBH_3_CN [10], to give compounds **7** and **8**. Additional aliquots of the Fmoc intermediate **5** were deprotected and then treated with *N,N*-di(Boc)-*S*-methylisothiourea, HBTU and HBPyU in the presence of a base to provide the corresponding *N*-guanylated derivatives **9-11** [11,12]. Finally, resin-bound derivatives **7-11** were cleaved from the resin with TFA:H_2_O (19:1, v/v) to render the corresponding *N*-alkyl (**17, 18**) and *N*-guanidyl derivatives **19-21** in low-moderate yield and high purity after extraction in reverse-phase cartridges. When the same reactions were applied to dipeptidyl resin **6**, after Alloc removal, the expected *N*^ε^-benzyl and *N*^ε^-isobutyl derivatives **22a, 23a** were obtained after cleavage. However, guanidylation reactions mainly resulted in the corresponding diguanidylated compounds **24b-26b**. A plausible explanation could be that the free amino group of the Lys side-chain, generated after removal of the Alloc group, could be basic enough under the guanidylation reaction conditions to promote the Fmoc removal in intermediate **6**.

As reported by Farrera-Sinfreu *et al.* [13], this undesired removal of the Fmoc group from Lys peptides seems to be time- and base-dependent, and could be prevented by quick chemical capture of the ε-NH_2_ group, not applicable in our case. Accordingly, the treatment with *N,N*-di(Boc)-*S*-methylisothiourea, requiring the presence of mercury (II) chloride as catalyst and TEA and repeated twice for reaction completion [11], the only isolated product was the disubstituted compound **24b**. However, guanidylation using HBTU or HBPyU in the presence of DIEA led to inseparable mixtures of mono- and di-substituted derivatives, **25a + 25b** and **26a + 26b** in a ~1:3 ratio (Scheme 1, Table 1). All compounds were obtained as single diastereoisomers, as deduced by HPLC and ^1^H-NMR, indicating the stereochemical integrity of all the steps in the process.

**Table 1:** Final compounds and intermediates prepared.

**Compound**	R^1^	**Compound**	R^1^	R^2^
**7, 17**	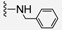	**12, 22**	–	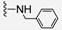
**8, 18**	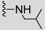	**13, 23**	–	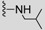
**9, 19**	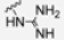	**14b, 24b**	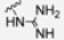	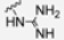
**10, 20**	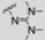	**15a, 25a**	–	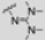
**11, 21**	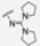	**15b, 25b**	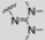	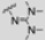
		**16a, 26a**	–	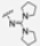
		**16b, 26b**	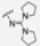	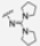

### 2.2. Biological activity

**Figure 1:**
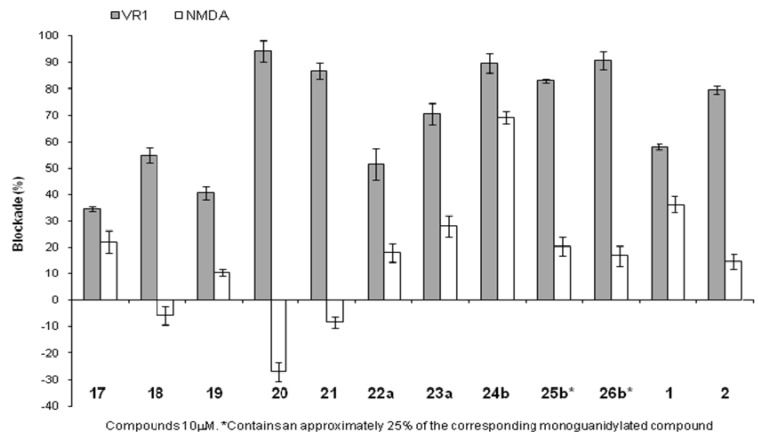
Blockage of TRPV1 and NMDA activity by compounds **17-26**. Each bar represents the mean ± SEM, *n* = 12.

Compounds **17-26** were evaluated for their ability to inhibit Ca^2+^ influx through TRPV1 channels, activated by capsaicin, and to block the glutamate-evoked activity of NMDA receptors (Figure 1). Model compounds **1** and **2** were included for comparative purposes. As shown in Figure 1, the incorporation of alkyl groups at the *N*–terminus of Trp residue decreases (*^i^*Bu, **17**) or maintains (Bzl, **18**) the TRPV1 blockage with respect to the parent compound H-Trp(Nps)-Lys-NH_2_ (**1**). A free guanidinium moiety at this position also reduces the TRPV1 antagonist properties, while the incorporation of tetramethyl- and pyrrolidyl-substituted guanidinos improves the percentage of TRPV1 blockage (**20** and **21**). It is noteworthy that, in general, substitution at the *N*–terminus leads to better TRPV1/NMDA selectivity than the model dipeptide derivative.

*N*^ε^-Alkyl Lys derivatives **22a** and **23a** show comparable and slightly better activity and enhanced selectivity with respect to the Lys-model dipeptide **1**. Compared to the H-Trp(Nps)-Arg-NH_2_ (**2**) prototype, diguanidino derivatives **24b-25b** do not result in significant improvement of the blocking effects. While they either retain or slightly improve TRPV1 blockade, selectivity towards NMDA diminished, especially for compound **24b**, with two free guanidino groups.

## 3. Experimental

### 3.1. General

All reagents were of commercial quality. Solvents were dried and purified by standard methods. ^1^H- NMR spectra were recorded at 300 MHz on a Varian XL-300 instrument (samples at 5−10 mM concentrations). Electrospray mass spectra (ES-MS) were performed, in positive ion mode, on a Hewlett-Packard 1100SD or an HPLC-MS WATERS equipment, the last integrated by a 2695 separation module, a 2996 photodiode array detector and a Micromass ZQ 2000 spectrometer. Analytical HPLC chromatograms were done on a C_18_ reverse-phase column (SunFire C_18_, 4.6 × 50 mm; Flow rate: 1 mL/min; Gradient: 10% A to 50% A in 8 min, were A: CH_3_CN + 0.08% formic acid and B: H_2_O + 0.1% formic acid). The solid-phase syntheses were carried out manually, on plastic syringes with a porous filter, and attached to a vacuum manifold for fast removal of excess of reagents and solvent. Resins were swollen in DCM/DMF/DCM/DMF (4 × 0.5 min).

### 3.2. General procedure for the synthesis of Fmoc-Trp(Nps)-Lys(Boc or Alloc)-NH-PS (5, 6)

Previously swollen Rink amide-MBHA resin (1.0 g, 0.46 mmol) was treated with 20% piperidine in DMF for 30 min to remove the Fmoc group. After washing with DMF and DCM, the corresponding amino acid, *N*-Fmoc-L-Lys(Boc)-OH (0.32 g, 0.69 mmol) or *N*-Fmoc-L-Lys(Alloc)-OH (0.31 g, 0.69 mmol), HOBt (0.93 g, 0.7 mmol) and DIC (0.1 mL, 0.7 mmol) in anhydrous DMF (3 mL) were added. Couplings were allowed to proceed at room temperature overnight. The resins were washed repeatedly with DMF and DCM (5 × 0.5 min each). Then, the Fmoc group was removed by the above described treatment, and a second coupling with *N*-Fmoc-L-Trp-OH (0.29 g, 0.69 mmol), HOBt (0.93 g, 0.69 mmol) and DIC (0.1 mL, 0.69 mmol) in anhydrous DMF (3 mL) was performed. After coupling completion (as assessed by the nynhidrin test), the resins were washed with anhydrous DMF, and a solution of Nps-Cl (0.23 mg, 1.118 mmol) in AcOH:DMF (8:2, 2 mL) was added under Ar. The mixture was allowed to stand for 3 h at r.t., and then repeatedly washed with DMF and DCM (5 × 0.5 min each).

### 3.3. Typical procedure for Alloc removal

To the pre-swollen resin, under Ar atmosphere, dried DCM (1 mL) and phenylsilane (25 equiv.) were added. After 5 min, a solution of Pd[PPh_3_]_4_ (0.1 equiv.) in dried DCM (1 mL) was added and the mixture was allowed to stand for 1 h 30 min. After this time the resin was drained off, washed with DCM (5 x 1 min) and the deprotection procedure was repeated once more, allowing the mixture to stand for 30 min. Finally, the resin was successively washed with DCM/dioxane-H_2_O (9:1)/DMF/DCM (5 × 1 min).

### 3.4. Reductive amination

After removal of the Fmoc (compound **5**) or Alloc (compound **6**) protecting groups, the resulting resins (0.11 mmol) were washed with DMF (5 × 0.5 min), and trimethyl orthoformate (TMOF) (2 × 0.5 min). Then, TMOF (0.5 mL) and the desired aldehyde (benzaldehyde or isobutyraldehyde, 0.33 mmol) were added. Formation of the imine at r.t. for 6 h was followed by filtration to remove the excess of aldehyde, addition of TMOF (1 mL), and addition of NaBH_3_CN (0.33 mmol), in the presence of AcOH 1% (0.01 mL) for 16 h.

### 3.5. Guanidylation reactions

#### 3.5.1. *N,N*-di(Boc)-*S*-methylisothiourea as reagent

To the previously swollen resins **5** and **6** (0.11 mmol), and after removal of Fmoc or Alloc groups, respectively, a solution of *N,N*-di(Boc)-*S*-methylisothiourea (0.13 mmol) in anhydrous DCM (1 mL) was added under Ar. Then, HgCl_2_ (0.22 mmol) and TEA (0.33 mmol) were successively added under Ar, and the reaction allowed to stand at r.t. overnight. This process was repeated once and then, the resins were drained and washed [DCM/DMF/DMF:MeOH (1:1.5)/DMF/DCM (5 × 0.5 min each)].

#### 3.5.2. HBTU or HBPyU as reagent

After removing the Fmoc and Alloc group from resins **5** and **6** (0.11 mmol), a solution of HBTU (0.275 mmol) or HBPyU (0.33 mmol) and DIEA (0.275 or 0.33 mmol, respectively) in anhydrous DMF (1 mL) was added, and the reaction allowed to stand at r.t. overnight. The resins were drained and washed [DMF /DCM/DMF/DCM (5 × 0.5 min each)].

#### 3.5.3. Cleavage reactions

For resins **12-16,** the removal of the Fmoc protecting group before cleavage was required. Resins **7-11** and **12-16** were treated with a mixture of TFA-H_2_O, 19:1 (1 mL for 100 mg) at r.t. for 2 h. After washings with DCM (5 × 1mL), the filtrates were evaporated and the residue solved in water and washed with ethyl acetate. The aqueous phase was lyophilized to give derivatives **17-26**, isolated as trifluoroacetate salts. When required, the products were purified through reverse-phase cartridges using mixtures of H_2_O/ACN as solvent.

*^i^Bu-Trp(Nps)-Lys-NH_2_* (**17**): Yield: 4%. HPLC: *t_R_* = 2.76 min. ^1^H-NMR (D_2_O):, δ 0.77 (d, 6H, 2CΗ_3_ *^i^*Bu, *J =* 6.6), 0.98 (m, 2H, γ−Η Lys), 1.3-1.4 (m, 4H, β and δ−H Lys), 1.81 (sept, 1H, CH *^i^*Bu, *J =* 6.7 Hz), 2.5-2.8 (m, 4H, ε−Η Lys and CH_2_*^i^*Bu), 3.2-3.3 (m, 2H, β−H Trp), 3.87-3.94 (m, 2H, α−H Lys and α−H Trp), 6.47 (d, 1H, Η_6_Nps, *J* = 7.8 Hz), 7.01 (t, 1H, H_5_ Indole, *J =* 7.2 Hz), 7.1-7.2 (m, 3H, H_6_ Indole, H_4_ and H_5_ Nps), 7.29 (d, 1H, H_7_ Indol, *J =* 8.1 Hz), 7.42 (d, 1H, H_4_ Indole, *J =* 8.8 Hz), 8.1 (d, 1H, H_3_ Nps, *J =* 7.8 Hz). Calc. MW = 540.68, MS ESI^+^ [M+H]^+^ m/z: 541.

*Bn-Trp(Nps)-Lys-NH_2_* (**18**): Yield: 24%. HPLC: *t_R_* = 3.42 min. ^1^H-NMR (D_2_O):, δ 1.03 (m, 2H, γ−Η Lys), 1.3-1.5 (m, 4H, β and δ−H Lys), 2.69 (t, 2H, ε−Η Lys, *J =* 7.6 Hz), 3.20 (m, 2H, β−H Trp), 3.94 (t, 1H, α−H Lys, *J =* 7.0 Hz), 4.02 (t, 1H, α−H Trp, *J =* 6.0 Hz), 4.60 (m, 2H, CH_2_ Bn), 6.44 (dd, 1H, Η_6_Nps, J=7.8 and 1.2 Hz), 7.02 (t, 1H, H_5_ Indole, *J =* 7.0 Hz), 7.1-7.3 (m, 9H, H_6_ and H_7_ Indole, H_4_ and H_5_ Nps, and 5H Ph), 7.44 (dd, 1H, H_4_ Indole, *J =* 7.9 and 0.75 Hz), 8.1 (dt, 1H, H_3_ Nps, *J =* 7.8 and 1.5 Hz). Calc. MW = 574.69, MS ESI^+^ [M+H]^+^ m/z: 575.

*[H_2_N-C(=NH)]-Trp(Nps)-Lys-NH_2_* (**19**): Yield: 36%. HPLC: *t_R_* = 2.41 min. ^1^H-NMR (D_2_O): δ 1.07 (m, 2H, γ−Η Lys), 1.3-1.5 (m, 4H, β and δ−H Lys), 2.70 (t, 2H, ε−Η Lys, *J =* 7.5 Hz), 3.2-3.3 (m, 2H, β−H Trp), 3.99 (t, 1H, α−H Lys, *J =* 7.05 Hz), 4.34 (t, 1H, α−H Trp, *J =* 5.8 Hz), 6.45 (d, 1H, Η_6_Nps, *J* =7.8 Hz), 7.01 (t, 1H, H_5_ Indole, *J =* 7.5 Hz), 7.1-7.2 (m, 3H, H_6_ Indole, H_4_ and H_5_ Nps), 7.28 (d, 1H, H_7_ Indole, *J =* 8.4 Hz), 7.43 (d, 1H, H_4_ Indole, *J =* 8.1 Hz), 8.1 (d, 1H, H_3_ Nps, *J =* 7.8 Hz). Calc. MW = 526.61, MS ESI^+^ [M+H]^+^ m/z: 527.

*[[(CH_3_)_2_N]_2_-C=]-Trp(Nps)-Lys-NH_2_* (**20**): Yield: 35%. ^1^H-NMR (D_2_O): δ 1.11 (m, 2H, γ−Η Lys), 1.4-1.5 (m, 4H, β and δ−H Lys), 2.69-2.77 (m, 14H, 2 ε−Η Lys and 4CH_3_-N), 3.22 (m, 2H, β−H Trp), 3.95 (t, 1H, α−H Lys, *J =* 6.9 Hz), 4.24 (t, 1H, α−H Trp, *J =* 7.8 Hz), 6.54 (d, 1H, Η_6_Nps, *J* = 7.8 Hz), 7.07 (t, 1H, H_5_ Indole, *J =* 7.3 Hz), 7.1-7.3 (m, 3H, H_6_ Indole, H_4_ and H_5_ Nps), 7.35 (d, 1H, H_7_ Indol, *J =* 8.4 Hz), 7.52 (d, 1H, H_4_ Indole, *J =* 8.1 Hz), 8.1 (dd, 1H, H_3_ Nps, *J =* 8.2 and 1.5 Hz). Calc. MW = 582.72, MS ESI^+^ [M+H]^+^ m/z: 583.

*[[(C_4_H_8_)_2_N]_2_-C=]-Trp(Nps)-Lys-NH_2_* (**21**): Yield: 47%. HPLC: *t_R_* = 3.79 min. ^1^H-NMR (D_2_O): δ 1.05 (m, 2H, γ−Η Lys), 1.3-1.5 (m, 4H, β and δ−H Lys), 1.62 (s, 8H, CCH_2_C Pyrrolidino), 2.71 (t, 2H, ε−Η Lys, *J =* 7.5 Hz), 3.0-3.2 (m, 10H, 2 β−H Trp and 4CCH_2_N Pyrrolidino), 3.93 (t, 1H, α−H Lys, *J =* 6.9 Hz), 4.25 (t, 1H, α−H Trp, *J =* 6.7 Hz), 6.49 (d, 1H, Η_6_Nps, *J* = 7.5 Hz), 7.0 (t, 1H, H_5_ Indole, *J =* 7.6 Hz), 7.1-7.2 (m, 3H, H_6_ Indole, H_4_ and H_5_ Nps), 7.29 (d, 1H, H_7_ Indole, *J =* 7.8 Hz), 7.44 (d, 1H, H_4_ Indol, *J =* 7.8 Hz), 8.1 (d, 1H, H_3_ Nps, *J =* 8.1 Hz). Calc. MW = 634.79, MS ESI^+^ [M+H]^+^ m/z: 635.

*H-Trp(Nps)-Lys(^i^Bu)-NH_2_* (**22a**): Yield: 8%. ^1^H-NMR (D_2_O): δ 0.74 (d, 3H, 1CΗ_3_ *^i^*Bu, *J =* 6.9 Hz), 0.77 (d, 3H, 1CΗ_3_ *^i^*Bu, *J =* 7.2 Hz), 1.06 (t, 1H, γ−Η Lys, *J =* 7.3 Hz), 1.3-1.4 (m, 4H, β and δ−H Lys), 1.85 (m, 1H, CH *^i^*Bu), 2.6-2.9 (m, 4H, ε−Η Lys and CH_2_*^i^*Bu), 3.18-3.21 (m, 2H, β−H Trp), 3.94 (t, 1H, α−H Lys, *J =* 7.2 Hz), 4.01 (t, 1H, α−H Trp, *J =* 7.5 Hz), 6.45 (d, 1H, Η_6_Nps, *J* = 6.9 Hz), 7.03 (t, 1H, H_5_ Indole, *J =* 7.5 Hz), 7.1-7.2 (m, 3H, H_6_ Indole, H_4_ and H_5_ Nps), 7.31 (d, 1H, H_7_ Indole, *J =* 8.1 Hz), 7.45 (d, 1H, H_4_ Indole, *J =* 8.1 Hz), 8.1 (d, 1H, H_3_ Nps, *J =* 8.1 Hz). Calc. MW = 540.68, MS ESI^+^ [M+H]^+^ m/z: 541.

*H-Trp(Nps)-Lys(Bn)-NH_2_* (**23a**): Yield: 14%.^1^H-NMR (D_2_O): δ 1.04 (m, 2H, γ−Η Lys), 1.3-1.4 (m, 4H, β and δ−H Lys), 2.70 (t, 2H, ε−Η Lys, *J =* 7.6 Hz), 3.17 (m, 2H, β−H Trp), 3.9-4.0 (m, 2H, α−H Lys and α−H Trp), 4.60 (m, 2H, CH_2_ Bn), 6.45 (d, 1H, Η_6_Nps, J=7.8), 7.03 (t, 1H, H_5_ Indole, *J =* 7.0 Hz), 7.1-7.3 (m, 9H, H_6_ and H_7_ Indole, H_4_ and H_5_ Nps, and 5H Ph), 7.45 (d, 1H, H_4_ Indole, *J =* 8.1 Hz), 8.1 (dd, 1H, H_3_ Nps, *J =* 7.8 and 1.8 Hz). Calc. MW = 574.69, MS ESI^+^ [M+H]^+^ m/z: 575.

*[H_2_N-C(=NH)]-Trp(Nps)-Lys[C(=NH)-NH_2_]-NH_2_* (**24b**): Yield: 15%.^1^H-NMR (D_2_O): δ 1.08 (m, 2H, γ−Η Lys), 1.3-1.6 (m, 4H, β and δ−H Lys), 2.94 (t, 2H, ε−Η Lys, *J =* 6.9 Hz), 3.24 (t, 2H, β−H Trp, *J =* 6.9 Hz), 4.04 (t, 1H, α−H Lys, *J =* 6.9 Hz), 4.40 (t, 1H, α−H Trp, *J =* 6.1 Hz), 6.52 (d, 1H, Η_6_Nps, J=7.8 Hz), 7.07 (t, 1H, H_5_ Indole, *J =* 7.5 Hz), 7.1-7.3 (m, 3H, H_6_ Indole, H_4_ and H_5_ Nps), 7.35 (d, 1H, H_7_ Indol, *J =* 8.1 Hz), 7.51 (d, 1H, H_4_ Indole, *J =* 7.8 Hz), 8.2 (d, 1H, H_3_ Nps, *J =* 8.1 Hz). Calc. MW = 568.65, MS ESI^+^ [M+H]^+^ m/z: 569.

*[[(CH_3_)_2_N]_2_-C=]-Trp(Nps)-Lys[=C-[N(CH_3_)_2_]_2_]-NH_2_* (**25b**). Yield: 10% (as a 3:1 mixture with compound **25a**). HPLC: *t_R_* = 4.68 min **25b** and 3.33 min **25a**. ^1^H-NMR **25b** (D_2_O): δ 1.11 (m, 2H, γ−Η Lys), 1.1-1.4 (m, 4H, β and δ−H Lys), 2.74 (s, 12H, 4CH_3_-N), 2.95 (t, 2H, ε−Η Lys, *J =* 7.0 Hz), 3.2-3.3 (m, 2H, β−H Trp), 3.94 (t, 1H, α−H Lys, *J =* 7.2 Hz), 4.24 (t, 1H, α−H Trp, *J =* 7.2 Hz), 6.54 (d, 1H, Η_6_Nps, J=7.8 Hz), 7.07 (t, 1H, H_5_ Indole, *J =* 7.8 Hz), 7.1-7.3 (m, 3H, H_6_ Indole, H_4_ and H_5_ Nps), 7.35 (d, 1H, H_7_ Indole, *J =* 8.1 Hz), 7.52 (d, 1H, H_4_ Indole, *J =* 8.1 Hz), 8.1 (d, 1H, H_3_ Nps, *J =* 8.1 Hz). Calc. MW = 680.86 **25b** and MW = 582.72 **25a**, MS ESI^+^ [M+H]^+^ m/z: 681 (62%), 583 (20%).

*[[(C_4_H_8_)_2_N]_2_-C=]-Trp(Nps)-Lys[=C-[(C_4_H_8_)_2_N]_2_]-NH_2_* (**26b**). Yield: 15% (as a 3:1 mixture with compound **26a**). ^1^H-NMR **26b** (D_2_O): δ 1.07 (m, 2H, γ−Η Lys), 1.3-1.6 (m, 4H, β and δ−H Lys), 1.68 (s, 8H, CCH_2_C Pyrrolidino), 2.9-3.1 (m, 4H, ε−Η Lys and β−H Trp), 3.2 (s, 8H, 4CCH_2_N Pyrrolidino), 3.95 (t, 1H, α−H Lys, *J =* 7.05 Hz), 4.28 (t, 1H, α−H Trp, *J =* 7.8 Hz), 6.51 (dd, 1H, Η_6_Nps, *J* = 8.1 and 1,2 Hz), 7.02 (t, 1H, H_5_ Indole, *J =* 7.5 Hz), 7.1-7.2 (m, 3H, H_6_ Indole, H_4_ and H_5_ Nps), 7.30 (d, 1H, H_7_ Indole, *J =* 7.8 Hz), 7.47 (d, 1H, H_4_ Indole, *J =* 7.8 Hz), 8.1 (dd, 1H, H_3_ Nps, *J =* 7.9 and 1.3 Hz). Calc. MW = 785.01, **26b** and MW = 634.79, **26a**, MS ESI^+^ [M+H]^+^ m/z: 786 (36%), 635 (12%). .

### 3.6. Biological activity

*Recombinant rat TRPV1 and human NMDAR channel expression in Xenopus oocytes and channel blockade*: All the procedures have been described in detail elsewhere [6,14]. Briefly, capped cRNA for TRPV1 (kindly provided by Dr. David Julius, University of California, San Francisco), and the NR1 and NR2A subunits of the NMDA receptor was synthesized from linearized cDNA by using the mMESSAGE mMACHINE from AMBION (Texas). cRNA (0.2 mg mL^-1^) was microinjected (V = 50 nL) into defolliculated oocytes (Stage V and VI) as described. Oocytes were functionally assayed 2–4 days after cRNA injection. Whole-cell currents from rat TRPV1-injected oocytes were recorded in standard Ringer solution (10 mM HEPES, pH 7.4, 115 mM NaCl, 2.8 mM KCl, 2.8 mM BaCl_2_) with a two-microelectrode voltage-clamp amplifier at 20 ºC. TRPV1 channels were activated by application of 10 µM capsaicin in the absence or presence of compounds (10 µM) at a holding potential (Vh) of −60 mV. Whole-cell currents from oocytes injected with NR1/NR2A (1:3, w/w) subunits were recorded in standard Ringer solution upon activation with L-glutamate (100 µM) supplemented with glycine (10 µM) at Vh = −60 mV. Responses were normalized with respect to that evoked without channel blockers. Experiments were performed in triplicate; each bar in Figure 1 represents the mean ± SEM, *n* (number of oocytes) = 12.

## 4. Conclusions

Convenient solid-phase procedures have been developed for the modification of Trp(Nps)-Lys dipeptides at the *N*-terminus or on the Lys side-chain. Main steps of these procedures are the AcOH-catalyzed incorporation of the Nps moiety at the indole ring, compatible with TFA labile resins, the reductive amination to *N*-alkyl derivatives, and the guanidylation reactions with thioureas or uronium salts. Diguanidylated by-products were obtained during the incorporation of guanidino groups on the Lys side-chain, probably due to Fmoc-removal by the free Lys ε–NH_2_ group under the basic media. From the biological point of view, the presence of substituted guanidino groups at *N*-terminus led to the best TRPV1 activity/NMDA selectivity ratio. Based on the significant activation of NMDA receptor by compound **20** (~30%), derivative **21** could be considered as a suitable molecule for further modifications in the search for new TRPV1 channel blockers.
